# The role of lipopolysaccharide as a marker of immune activation in HIV-1 infected patients: a systematic literature review

**DOI:** 10.1186/1743-422X-9-174

**Published:** 2012-08-27

**Authors:** Matteo Vassallo, Patrick Mercié, Jacqueline Cottalorda, Michel Ticchioni, Pierre Dellamonica

**Affiliations:** 1 Department of Infectious Diseases, L’Archet Hospital, University of Nice, Nice, France; 2 Department of Internal Medicine, University of Bordeaux, Bordeaux, France; 3 Department of Virology, L’Archet Hospital, University of Nice, Nice, France; 4 Department of Immunology, L’Archet Hospital, University of Nice, Nice, France

## Abstract

**Background:**

Recent observational studies suggest a role for lipopolysaccharide (LPS) as a marker of immune activation in HIV-infected patients, with potential repercussions on the effectiveness of antiretroviral regimens.

**Object:**

A systematic review of LPS as a marker of immune activation in HIV-1 infected patients.

**Data sources:**

MEDLINE register of articles and international conference proceedings.

**Review methods:**

Case–control studies comparing the role of plasma LPS as a marker of immune activation in HIV-infected patients versus HIV negative subjects.

**Data synthesis:**

Two hundred and six articles were selected using MEDLINE, plus 51 studies presented at international conferences. Plasma LPS is a marker of immune activation in HIV-infected patients, determining the entry of central memory CD4+ T cells into the replication cycle and finally generating cell death. Plasma LPS probably results from immune-mediated alterations of the intestinal barrier, which can occur soon after HIV seroconversion. LPS is a likely marker of disease progression, as it drives chronic monocyte activation, and some studies suggest that hyperexpression of CCR5 receptors, related to LPS plasma levels, could be responsible for monocyte trafficking in the brain compartment and for the appearance of HIV-associated neurocognitive disorders. Long-term combination antiretroviral therapy (cART) generally reduces LPS concentrations, but rarely to the same levels as in the control group. This phenomenon probably depends on ongoing but incomplete repair of the mucosal barrier. Only in patients achieving maximal viral suppression (i.e. viral load < 2.5 cp/ml) are LPS levels comparable to healthy donors. In successfully treated patients who did not restore CD4+ T cells, one hypothesis is that the degree of residual microbial translocation, measured by LPS, alters the turnover of CD4+ T cells.

**Conclusions:**

LPS is a marker of microbial translocation, responsible for chronic immune activation in HIV-infected patients. Even in successfully treated patients, LPS values are rarely normal. Several studies suggest a role for LPS as a negative predictive marker of immune restoration in patients with blunted CD4 T cell gain.

## Background

HIV-1 infection develops with acute viræmia and rapid depletion of CD4 T cells within mucosal-associated lymphoid tissues (MALT), particularly in gut lymphoid compartments [1].

Lipopolysaccharide (LPS) is a component of the cell wall of gram-negative bacteria and recent data show that plasma LPS reflects microbial translocation in HIV-infected patients [2]. Indeed, HIV-induced disruption of MALT results in translocation of microbial products across the intestinal mucosa into the peripheral circulation, producing high levels of plasma LPS and bacterial DNA that persist throughout chronic HIV infection [1,3].

The degree of microbial translocation has been associated with HIV progression [4,5]. Despite successful virological control under combination antiretroviral therapy (cART), some patients do not restore their cellular immunity and certain authors suggest a possible role for microbial translocation in persistent CD4T-cell depletion [6].

Our aim was to review the literature concerning the role of plasma LPS as an immune activator in HIV-infected patients and the impact of cART on LPS plasma levels.

## Methods

We obtained relevant articles from the Pubmed Mesh database, using the broad search terms “Lipopolysaccharides”, “HIV” and “Humans”.

We included studies regardless of date, language or publication status.

In addition, we searched abstracts from the last three Conferences on Retroviral and Opportunistic Infections (CROI), i.e. 2010, 2011 and 2012, since interest in LPS as an immune marker has been increasing over the past three years.

Inclusion criteria were case–control studies evaluating LPS plasma values in HIV-infected patients, compared to those in healthy controls.

We selected 206 articles from Pubmed and 51 abstracts from the past three CROI meetings describing the effect of LPS on the human immune system or the impact of cART on LPS.

**Figure 1:**
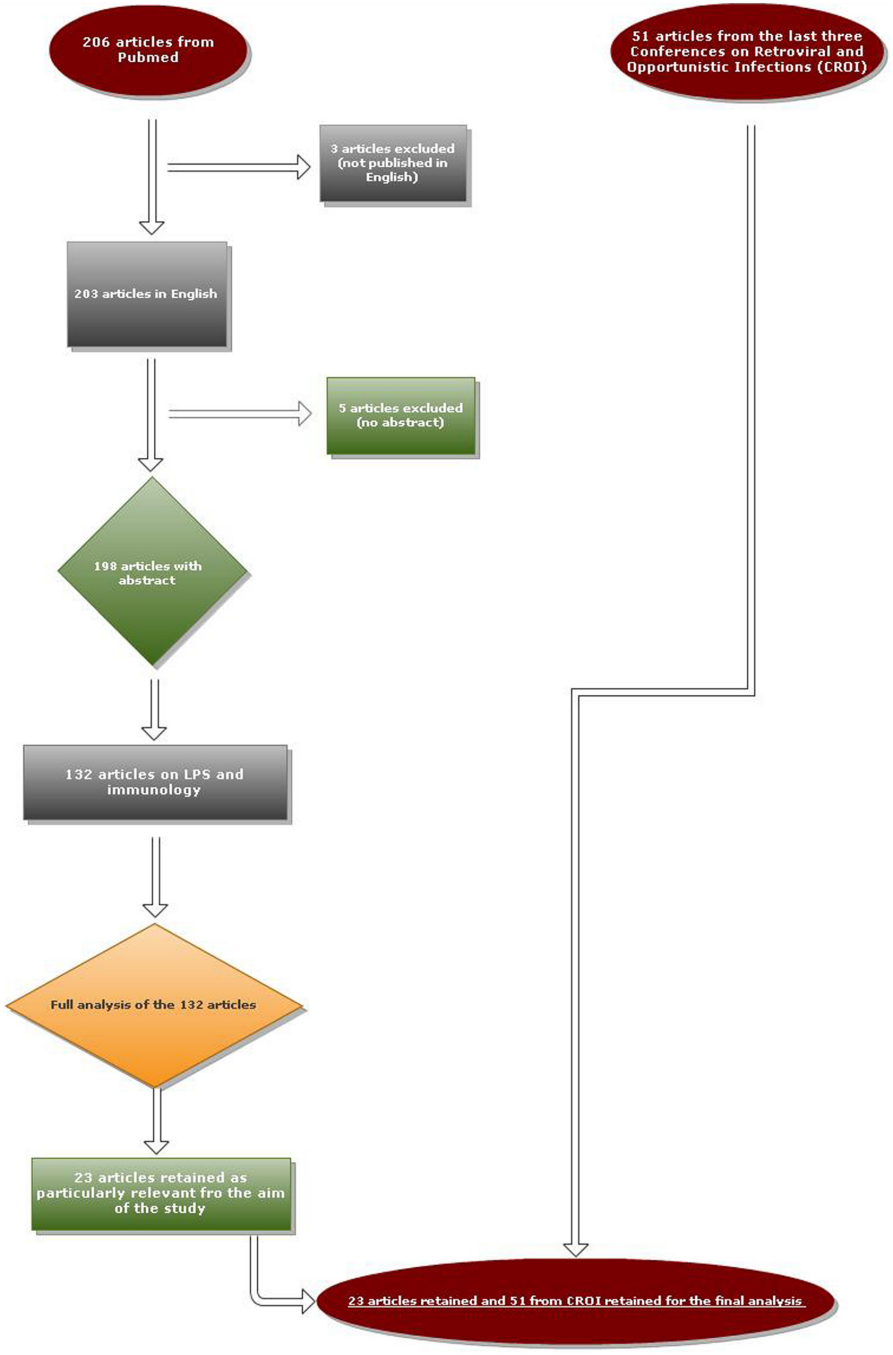
Selection of articles.

Among the 206 articles selected in Pubmed, 203 were written in English, 198 included an abstract and 132 focused more specifically on the immunological mechanisms related to LPS. After examining these 132 articles in full text, we retained 23 articles for their potential interest in focusing more specifically on LPS as a marker of immune activation or on the impact of cART on LPS. The remaining articles were excluded as their content did not provide further information with regard to the selected papers or were not considered relevant. Figure 1 summarizes the selection criteria for the articles.

### Ethical approval

Wallet et al.: The protocol was approved by University of Florida and University of South Florida/All Children’s Hospital Institutional Review Boards.

Brenchley et al.: All human subjects gave informed consent and all studies were approved by the appropriate Institutional Review Boards or Animal Care and Use Committees.

Redd et al.: Institutional Review Board approvals were obtained from the Uganda National Council for Science and Technology, and the Institutional Review Boards of collaborating U.S. Institution (Walter Reed Army Institute of Research, Columbia University, and John Hopkins University).

Estes et al.: Animals were housed and cared in accordance with American Association for Accreditation of Laboratory Animal Care standards in AAALAC accredited facilities, and all animal procedures were performed according to protocols approved by the Institutional Animal Care and Use Committees of the National Cancer Institute, California National Primate Research Center or Yerkes National Primate Research Center.

Jiang et al.: Theses studies were approved by the institutional review boards at the University Hospitals of Cleveland, the National Institutes of Health, and the University of California, San Francisco (UCSF), as well as at participating AIDS Clinical Trials Unit sites.

Papasavvas et al.: Informed consent was obtained according to the Human Experimentation Guidelines of the US Department of Health and Human Services and of the authors’ institutions. The study was approved by the Institution Review Boards of the Wistar Institute and Philadelphia FIGHT.

Munsaka et al.: The study was reviewed and approved by the University of Hawaii Review Board.

Balagopal et al.: All subjects provided informed consent for testing through a protocol approved by the Committees on Human Research of the John Hopkins School of Medicine or Bloomberg School of Public Health.

For the other experimental researches cited in the text, all patients provided written informed consent for use of their blood samples under local institutional review board approval.

## Results

### Increase of plasma LPS in HIV infected patients

Circulating LPS is likely to originate in the gastrointestinal (GI) tract [2,7]. Epple et al. [8] compared gastrointestinal mucosal barrier function among 11 untreated, 8 suppressively-treated HIV-infected patients, and 9 HIV-seronegative controls, and found that HIV infection induces intestinal barrier defect by mucosal CD4 T-cell depletion, villous atrophy and alteration of tight-junction protein composition. These alterations are mainly cytokine-mediated, particularly via IL2, IL4 and TNF-alpha.

Estes et al. showed that microbial translocation is associated with a breakdown of integrity of the intestinal epithelial barrier of simian immunodeficiency virus (SIV) infected rhesus macaques (RMs) [9]. The extent of mucosal damage was correlated to the extent of microbial translocation, thus generating the production of inflammatory cytokines and a dysfunction in the ability of macrophages to phagocytose translocated microbial products. This phenomenon differs from other natural hosts of SIV, such as African green monkeys (AGMs) and Sooty mangabeys (SMs), who do not progress to AIDS and in whom immune activation is minimal. Indeed, authors found neither epithelial barrier breakdown nor microbial products in the *lamina propria* of chronically SIV-infected AGMs and SMs [9].

It is likely that mucosal damage in the gastrointestinal tract of SIV-infected RMs and HIV-infected patients leads to levels of microbial translocation that exceed the capacity of host defense mechanisms, generating a status of persistent immune activation.

### Effects of chronic LPS stimulation in vivo

Plasma LPS triggers monocyte/macrophage activation, generating the production of soluble CD14 (sCD14) and pro-inflammatory cytokines (TNF, IL-1). The correlation between plasma LPS levels and the frequency of circulating CD8 T-cells with an activated CD38+ HLA-DR + phenotype suggests that microbial translocation might directly, or indirectly via cytokines and chemokines, generate polyclonal T-cell activation [2].

In a cross-sectional study comparing 227 HIV-infected patients to 15 controls, Jiang et al. [10] showed that HIV infection results in higher plasma levels of bacterial products (LPS and bacterial 16S rDNA) and that these values were correlated to T-cell activation markers (CD38 and HLA). The authors suggest a model in which bacterial products generate heightened expression of CD38 and HLA and movement of central memory CD4+ T cells into the cell cycle, finally resulting in cell death.

Indeed, Funderburg et al. recently showed that changes in sCD14 levels correlate with changes in CD4 and central memory CD4 T-cell cycling, suggesting that microbial translocation may play a role in the turnover of central memory CD4 cells in HIV infection [11].

Several studies [2,12,13] confirmed that LPS is a marker of T-cell activation. D’Ettorre et al. [14] recently showed that, in cART–treated subjects, the level of HIV-DNA in the gut mucosa is correlated with levels of LPS and CD8 + CD38+ T cells.

Some articles fail to show a correlation between LPS and immuno-virological markers [1,15]. Wallet et al., for example, conducted a longitudinal study on 14 healthy and 33 perinatally HIV-1 infected patients, in whom LPS levels were linked to monocyte/macrophage activation markers but not to T-cell activation. The authors suggest that microbial translocation affects monocyte/macrophage activation independently of lymphocyte activation and HIV viral load.

Moreover, two recent works did not show any relationship between microbial translocation and T-cell immune activation, in HIV-infected children on successful cART for Pilakka-Kanthikeel et al. [16] and in patients interrupting treatment for Papasavvas et al. [17].

HIV elite controllers (EC), defined as patients with undetectable viral load (< 75 cp/mL) in the absence of antiretroviral treatment, have higher plasma LPS and CD8+ T-cell activation levels than non-infected subjects, suggesting that the early loss of CD4+ T cells from the *lamina propria* of the gastrointestinal tract and the physical disruption of the mucosal barrier may contribute to generalized T-cell activation [12].

Lopez et al. [13] found that LPS in EC is positively correlated with the activation of central-memory CD8+ T-cells and negatively with central-memory CD4+ T cells, differing from successfully treated (ST) patients and arguing against an indefinitely benign clinical status of EC.

### Innate mechanisms to decrease effects of microbial translocation

In order to reduce the inflammatory response due to microbial translocation, at least two kinds of mechanisms exist: first, clearance of LPS by the so-called endotoxin-core antibodies (EndoCAb), which are produced by T-dependent B-cells and play a major role, especially during acute microbial translocation such as sepsis, by reducing the amplitude of the inflammatory response. EndoCab levels are also increased in chronic microbial translocation, as occurs in inflammatory bowel disease as a part of humoral response to LPS, but in chronic HIV infection their levels are insufficient to neutralize circulating LPS and prevent systemic immune activation, probably as a consequence of B-cell dysfunction. Brenchley et al. found that EndoCAb levels were higher in uninfected individuals and in acute/early infected subjects than in HIV-infected progressors [2].

The second mechanism to reduce the inflammatory response is the functional impairment in monocytes, generated by repeated stimulation of these cells by LPS*.* This monocytic state is not a complete deactivation but rather a specific reorientation in the reaction of the cells to LPS, resulting in reduced production of pro-inflammatory cytokines [18,19]. Typically seen during acute conditions, e.g. sepsis, trauma or major surgery, it can also be observed during chronic infections, as shown by Brenchley et al., who found in HIV-infected patients a significant inverse correlation between the ability of monocytes to respond in vitro to LPS stimulation and plasma levels of LPS [2].

### Clinical consequences of immune activation

A study by Ancuta et al. suggests a role for elevated LPS levels in driving monocyte activation in HIV disease progression [20], while Munsaka et al. [21] showed that LPS-stimulation of monocytes in HIV-negative patients augmented surface CCR5 expression from 21% to 99%, increasing the risk for non-infected monocytes to become infected.

Marchetti et al. showed that circulating LPS during the initial years of chronic HIV infection is a strong predictor of disease progression independently of CD4 cell count and HIV viræmia, suggesting its role as a biomarker for HIV disease monitoring [22].

Several studies showed that monocytes are the key cells in the pathogenesis of HIV-associated neurocognitive disorders, so that the hyper-expression of CCR5 induced by LPS could have potential consequences also in HIV-infected patients if confirmed by other studies. Indeed, in another study by Ancuta et al., plasma LPS levels were higher in patients with HIV-associated dementia, probably as a consequence of increased monocyte trafficking in the brain [7].

Moreover, elevated LPS plasma levels increase the risk of progression to chronic liver disease in hepatitis C-infected patients, as LPS binds to Kupfer cells, up-regulating the expression of proinflammatory and profibrogenic cytokines such as tumor necrosis factor alpha (TNFα), interleukin 1(IL-1), IL-6 and IL-12 [23].

Bellistri et al. [24] showed that increased microbial translocation is associated with a lack of early virological response to HCV therapy in HIV-HCV co-infected patients.

Microbial translocation and macrophage activation appear also to raise cardiovascular risks, as suggested by Keledis et al., who showed that LPS and sCD14 are associated with increases in the yearly rate of change in carotid-intima thickness in HIV + patients [25]. Moreover, Fundenburg *at al.* showed that LPS exposure increases procoagulant tissue factor expression on monocytes of HIV-infected patients, suggesting a contribution to coronary artery disease [26]. The main exception is the study conducted by Redd et al., who did not find any correlation between microbial translocation and disease progression in a longitudinal cohort study of HIV-1-infected African subjects, contrary to the majority of American and European studies [3]. According to disease outcomes, the authors divided patients into long-term nonprogressors, standard progressors and rapid progressors, finding no differences in LPS and EndoCAb levels among these groups. One explanation suggested by the authors is that the mode of transmission may play a pivotal role, as studies examining microbial translocation in the United States or Europe concern male populations who most likely contracted the infection through homosexual contact or intravenous drug use, while the majority of African HIV-infected subjects are heterosexual. Among other possible explanations for these discordant results, it may be that different pre-infection levels of EndoCab are linked to environmental exposure to intestinal infections, or that different microbial translocation markers are a symptom and not a cause of disease progression [3].

### Effects of cART on microbial translocation

In general, long term cART has been associated with reduced plasma LPS and immune activation markers. However, LPS plasma levels often remain detectable in successfully treated patients on cART, contrary to healthy donors, as shown by Jiang et al. [10]. The authors suggest that this phenomenon may be explained by ongoing partial repair of the mucosal barrier.

Soon after initiation of cART (1, 8, and 48 weeks), microbial translocation, immune activation markers and plasma levels of HIV-RNA decrease consistently over time, whereas CD4+ T-cell numbers increase. However, repeated-measures analysis indicates that microbial translocation decrease is independent of changes in HIV-RNA levels [10]. Indeed, comparison between treated and untreated patients with comparable levels of viræmia showed that the first have lower levels of microbial translocation [10].

No association between microbial translocation and CD4 T-cell count was observed, neither 1 week nor 8 weeks after starting cART, suggesting that the relationship between microbial translocation and immune recovery is more closely related to the cellular turnover linked to chronic infection than to the initial rapid increase in circulating CD4 T-cells after the introduction of cART [10].

Baroncelli et al. [6] showed that microbial translocation is associated with residual viral replication. Indeed, comparison between 25 patients on cART with HIV viral load values between 2 and 50 copies/ml and 19 subjects with more complete suppression (i.e. < 2.5 cp/ml) showed that only the second group attained the same levels of LPS as HIV-uninfected subjects, suggesting that cART can revert the HIV-induced gastrointestinal barrier defect that is responsible for microbial translocation.

Of particular interest is the observation that treatment interruptions not exceeding two months in one study [6], three months in another [27], did not increase LPS plasma levels, in contrast with immune activation markers and viræmia.

**Figure 2:**
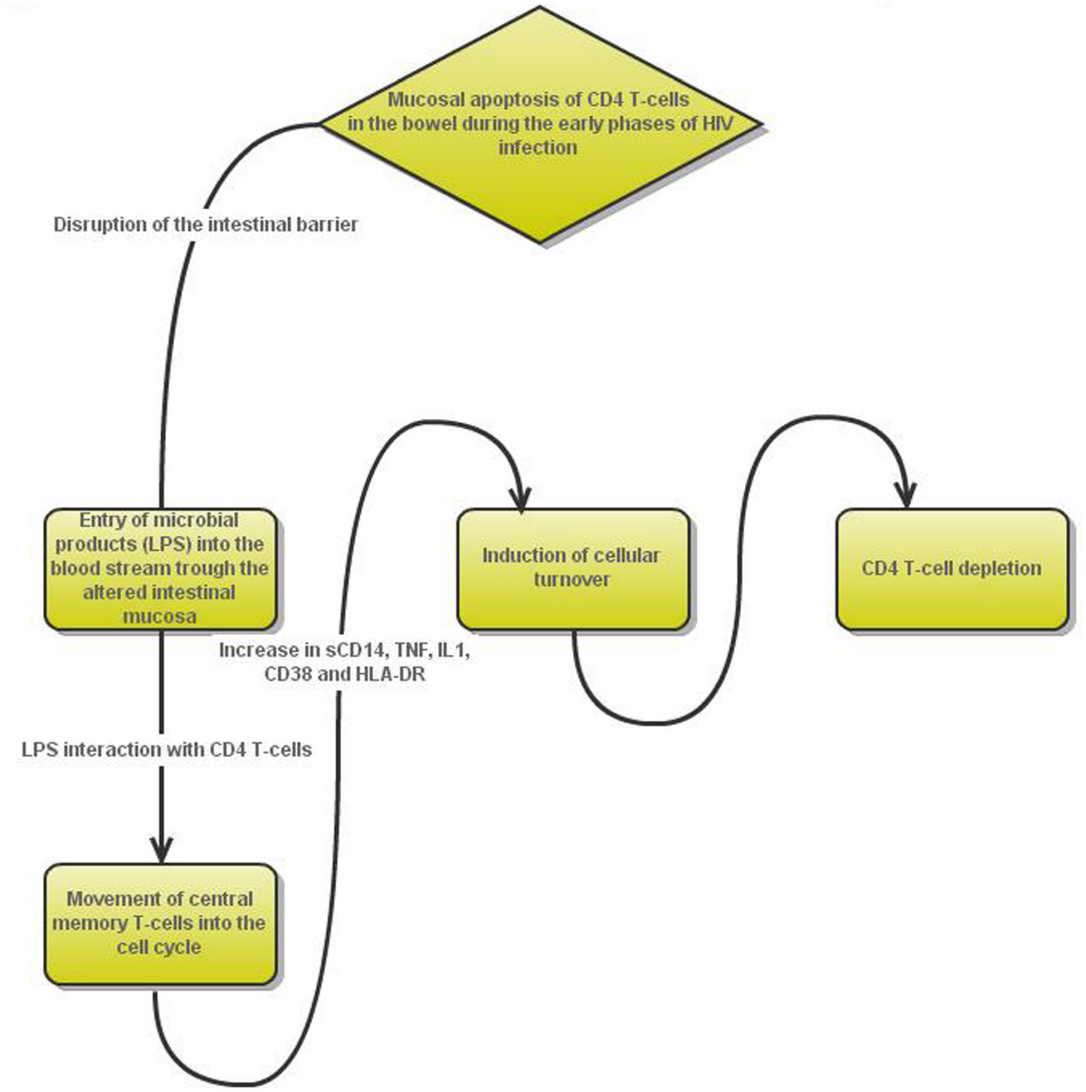
Microbial translocation and CD4 cell depletion.

In ST patients with blunted CD4+ T-cell gains, it has been suggested that chronic immune activation and increased cellular turnover, induced by such persistent microbial translocation via the mechanisms described above, might play a key role Figure 2[10,12]. 

Bandera et al. confirmed that immunological non responder patients have higher levels of LPS than full responders and a higher proportion of Th17 cells, which may be involved in the proinflammatory status of non responders [28]. Similarly, Estes et al. suggested that a preferential loss of GI tract Th-17 cells is linked to persistent immune activation [9].

Tables 1 and 2 summarize current knowledge on LPS.

**Table 1:** Microbial translocation and immunological outcomes in different populations of HIV-infected patients (from all the selected articles included in this review)

	**LPS plasma values compared to healthy donors**	**Main immunological outcomes**
Untreated HIV + patients	++++	Chronic CD4 + T cell death, potential risk of accelerated disease progression
HIV + elite controllers	+++	Unknown
HIV + successfully treated patients	+/=	Potential risk of for blunted CD4+ T cell gain. Only maximal virological suppression (< 2.5 cp/ml) allows normalisation of LPS values
HIV + on treatment, with persistent viral replication	++	Potential risk of blunted CD4+ T cell gain, to a higher extent than for patients with viral control

Elite controllers: viral load < 75 cp/ml without the effect of cART.

Successfully treated patients: viral load < 40 cp/ml on cART.

Patients with viral replication: viral load > 40 cp/ml, on cART.

**Table 2:** Current knowledge on lipopolysaccharides and HIV-infection

**Accepted statements concerning LPS**	**Main articles**
1) Plasma values of LPS reflect microbial translocation	Jiang et al. [10], Ancuta et al. [7], Brenchley et al [2].
2) LPS is a marker of cellular activation, and probably of T-cell activation	Hunt et al. [13], Jiang et al. [10]
3) Plasma values of LPS in HIV-infected patients derive from gut modification, which is largely dependent on mucosal cytokines	Epple et al. [8]
4) LPS levels generally decrease after initiation of cART, but rarely to the same values as in healthy subjects	Jiang et al. [10], Baroncelli et al. [6]
**Aspects of LPS that remain to be explained**	
1) Is microbial translocation a cause or a consequence of cellular activation?	
2) Is gut disruption completely reversible after initiating cART?	
3) Does LPS play a key role in virologically controlled patients with blunted CD4 cell gain?	

## Conclusions

Plasma LPS is a marker of microbial translocation in HIV-infected patients and its origin is probably a defective intestinal barrier, where damage begins during the early phase of HIV infection and continues during the chronic period.

Mucosal damage and macrophage phagocytic dysfunctional clearance of microbial products are responsible for microbial translocation in blood.

Several studies indicate that microbial translocation is associated with immune activation, with production of sCD14 and pro-inflammatory cytokines.

There is increasing evidence that broad immune activation is an important driver of disease pathogenesis and is responsible for CD4 T-cell depletion and HIV-related co-morbid conditions. The main exception is a study by Redd et al., who found little effect of microbial translocation on the rate of HIV-1 disease in African patients. Authors explain that these results could be due to the difference in patient characteristics, but they also suggest that microbial translocation could be a symptom and not a cause of disease progression.

Certain innate immune mechanisms counteract the effect of microbial translocation, e.g. production of antibodies to neutralize LPS activity and functional impairment of macrophages when chronically stimulated by LPS, but during HIV-infection these mechanisms are generally insufficient to prevent systemic immune activation.

The extent of microbial translocation is not correlated to the viral load, as shown for example by Hunt and Lopez, who found that even HIV EC display markers of immune activation, suggesting that aviremic patients also sustain GI tract damage that persists over time.

The introduction of cART reduces the degree of microbial translocation, but in general not to the levels observed among uninfected subjects. The mechanisms of LPS reduction when starting cART are probably not completely understood, as they do not depend on viral load, but come into play very soon after treatment initiation.

However, the lack of an association between reduced microbial translocation and increased CD4 cell count during the first weeks of treatment suggests that microbial translocation has a greater impact on the cellular turnover of latently infected cells than on circulating CD4 T-cells.

The persistence of detectable levels of plasma LPS in ST patients could be explained in at least three ways: first, intestinal barrier repair is only partially achieved with cART, suggesting that early treatment could be more effective. Second, only regimens achieving maximal viral suppression (i.e. < 2.5 cp/mL) allow complete normalisation of the mucosal barrier. Third, LPS decrease depends on complex immunological mechanisms, which are only partially understood.

However, regardless of the causes of persistently high LPS plasma levels in ST patients, there is increasing evidence that microbial translocation could be responsible for blunted CD4+ T-cell gains, by driving cellular turn-over.

These results suggest that, in case of blunted CD4+ T cell gains despite successful cART, LPS could be used as a negative predictor of immune restoration.

Further studies in primary HIV infection would be helpful to find out if early introduction of cART, perhaps with the use of the novel classes of antiretroviral agents (integrase and entry inhibitors), could reduce mucosal damage and avoid systemic immune activation during the chronic phase, thus preventing further impairment of immunological response.

## Competing interests

All authors declare that they have no competing interests.

## Authors’ contributions

MV participated in the study concept, acquisition, analysis and interpretation of data, and in the critical review of the manuscript. PM participated in the study concept, analysis and interpretation of data and critical review of the manuscript. JC, MT and PD participated in the critical review of the manuscript. All authors read and approved the final manuscript.

## References

[B1] Wallet MA Rodriguez CA Yin L Saporta S Microbial translocation induces persistent macrophage activation unrelated to HIV-1 levels or T-cell activation following therapy AIDS 2010 24 1281 1290 10.1097/QAD.0b013e328339e228 20559035 PMC2888494

[B2] Brenchley JM Price DA Schacker TW Asher TE Microbial translocation is a cause of systemic immune activation in chronic HIV infection Nat Med 2006 12 1365 1371 17115046 10.1038/nm1511

[B3] Redd A Dabitao D Bream J Charvat B Microbial translocation, the innate cytokine response, and HIV-1 disease progression in Africa Proc Natl Acad Sci USA 2009 106 6718 6723 10.1073/pnas.0901983106 19357303 PMC2667149

[B4] Marchetti G Cozzi-Lepri A Merlini E Bellistri GM Microbial translocation predicts disease progression of HIV-infected antiretroviral-naive patients with high CD4+ cell count AIDS 2011 25 1385 1394 10.1097/QAD.0b013e3283471d10 21505312

[B5] Nowroozalizadeh S Mansson F da Silva Z Repits J Microbial translocation correlates with the severity of both HIV-1 and HIV-2 infections J Infect Dis 2010 201 1150 1154 10.1086/651430 20199244

[B6] Baroncelli S Galluzzo CM Pirillo M Mancini MG Microbial translocation is associated with residual viral replication in HAART-treated HIV+ subjects with < 50 copies/ml HIV-1 RNA J Clin Virol 2009 46 367 370 10.1016/j.jcv.2009.09.011 19782638

[B7] Ancuta P Kamat A Kunstman KJ Kim E Microbial translocation is associated with increased monocyte activation and dementia in AIDS patients Plos One 2008 3 e2516 10.1371/journal.pone.0002516 18575590 PMC2424175

[B8] Epple HJ Schneider T Troeger H Kunkel D Impairment of the intestinal barrier is evident in untreated but absent in suppressively treated HIV-infected patients Gut 2009 58 220 227 10.1136/gut.2008.150425 18936106

[B9] Estes JD Harris LD Klatt NR Tabb B Pittaluga S Damaged intestinal epithelial integrity linked to microbial translocation in pathogenic simian immunodeficiency virus infections Plos Pathogens 2010 6 e1001052 10.1371/journal.ppat.1001052 20808901 PMC2924359

[B10] Jiang W Lederman MM Hunt P Sieg SF Plasma levels of bacterial DNA correlate with immune activation and the magnitude of immune restoration in persons with antiretroviral-treated HIV infection J Infect Dis 2009 199 1177 1185 10.1086/597476 19265479 PMC2728622

[B11] Funderburg N Andrade A Chan E Lu D Rosenkranz S Delayed reduction in CD4 T cell turnover following viral control correlates with markers of microbial trnaslocation in treatment-naive patients receiving RAL-based ART: preliminary results from ACTG A5248 2011 Boston: 18th Conference on Retroviruses and Opportunistic Infections February 2011, Boston, Session 28, poster 318

[B12] Hunt PW Brenchley J Sinclair E McCune JM Relationship between T cell activation and CD4+ T cell count in HIV-seropositive individuals with undetectable plasma HIV RNA levels in the absence of therapy J Infect Dis 2008 197 126 133 10.1086/524143 18171295 PMC3466592

[B13] López M Soriano V Peris-Pertusa A Rallón N Elite controllers display higher activation on central memory CD8 T cells than HIV patients successfully on HAART AIDS Res Hum Retroviruses 2011 27 157 165 10.1089/aid.2010.0107 20964478

[B14] D’Ettorre G Paiardini M Zaffiri L Andreotti M HIV persistence in the gut mucosa of HIV-infected subjects undergoing antiretroviral therapy correlates with immune activation and increased levels of LPS Curr HIV Res 2011 9 148 153 10.2174/157016211795945296 21457131

[B15] Cassol E Rossow T Seebregts C Cassol S Microbial translocation: a marker of advanced HIV-1 infection and a predictor of treatment failure? J Infect Dis 2011 203 747 748 10.1093/infdis/jiq109 21278212 PMC3072722

[B16] Pilakka-Kanthikeel S Huang S Fenton T Borkowsky W Microbial translocation is increased in HIV-infected children on ART and is independent of viral replication and immune activation18th Conference on Retroviruses and Opportunistic Infections 2011 142 728 Boston, Session

[B17] Papasavvas E Pistilli M Reynolds G Bucki R Delayed loss of control of plasma lipopolysaccharide levels after therapy interruption in chronically HIV-1 infected patients AIDS 2009 23 369 375 10.1097/QAD.0b013e32831e9c76 19114856 PMC2745273

[B18] Wolk K Docke WD von Baehr V Volk HD Sabat R Impaired antigen presentation by human monocytes during endotoxin tolerance Blood 2000 96 218 223 10891454

[B19] Porcheray F Viaud S Rimaniol AC Leone C Samah B Macrophage activation switching: an asset for the resolution of inflammation Clin Exp Immunol 2005 142 481 489 16297160 10.1111/j.1365-2249.2005.02934.x PMC1809537

[B20] Ancuta P Autissier P Wurcel A Zaman T Stone D Gabuzda D CD16+ monocyte-derived macrophages activate resting T cells for HIV infection by producing CCR3 and CCR4 ligands J Immunol 2006 176 5760 5771 16670281 10.4049/jimmunol.176.10.5760

[B21] Munsaka SM Agsalda M Troelstrup D Hu N Yu Q Shiramizu B Characteristics of activated monocyte phenotype support R5-tropic human immunodeficiency virus Immunol Immunogenetics Insights 2009 1 15 20 19997535 10.4137/iii.s2011 PMC2789571

[B22] Marchetti G Cozzi-Lepri A Merlini E Bellistri GM Castagna A Microbial translocation predicts disease progression of HIV-infected antiretroviral naive patients with high CD4+ cell count AIDS 2011 25 1385 1394 10.1097/QAD.0b013e3283471d10 21505312

[B23] Balagopal A Philp FH Astemborski J Block TM Human immunodeficiency virus-related microbial translocation and progression of hepatitis C Gastroenterology 2008 135 226 233 10.1053/j.gastro.2008.03.022 18457674 PMC2644903

[B24] Bellistri GM Pegorer P Tincati C Meroni L Microbial Translocation Is a Determinant of Persisting T Cell Hyperactivation in HIV-infected Patients Failing to Recover CD4 following Long-term HAART 2011 San Francisco: 17th Conference on Retroviruses and Opportunistic Infections February 2011, San Francisco, Session 11, poster 377

[B25] Keledis A Yang O Kendall M Hodis H Currier J Biomarkers of microbial translocation and macrophage activation are associated with progression of atherosclerosis in HIV infection: ACTG NWCS 332/A5078 study 2012 Seattle: Conference on Retroviruses and Opportunistic Infections March 2012, Seattle, Session 36, poster 122

[B26] Funderburg N Zidar D Shive C Lioi A Mudd J CD14Dim CD16+ monocytes are proportionally increased in HIV+ infected persons and express increased levels of the procoagulant tissue factor 2012 Seattle: 19th Conference on Retroviruses and Opportunistic Infections March 2012, Seattle, Session 67, poster 321

[B27] Papasavvas E Pistilli M Reynolds G Bucki R Azzoni L Delayed loss of control of plasma lipopolysaccharide levels after therapy interruption in chronically HIV-1-infected patients AIDS 2009 23 369 375 10.1097/QAD.0b013e32831e9c76 19114856 PMC2745273

[B28] Bandera A Trabattoni D Pinnetti C Muscatello A Cappelletti E Up-regulation of Th17 cells in HIV+ patients with low CD4 cell count on effective cART 2012 Seattle: 19th Conference on Retroviruses and Opportunistic Infections March 2012, Seattle, Session 68, poster 329

